# Is telemedicine the answer to rural expansion of medication treatment for opioid use disorder? Early experiences in the feasibility study phase of a National Drug Abuse Treatment Clinical Trials Network Trial

**DOI:** 10.1186/s13722-021-00233-x

**Published:** 2021-04-20

**Authors:** Yih-Ing Hser, Allison J. Ober, Alex R. Dopp, Chunqing Lin, Katie P. Osterhage, Sarah E. Clingan, Larissa J. Mooney, Megan E. Curtis, Lisa A. Marsch, Bethany McLeman, Emily Hichborn, Laurie S. Lester, Laura-Mae Baldwin, Yanping Liu, Petra Jacobs, Andrew J. Saxon

**Affiliations:** 1 Department of Psychiatry and Biobehavioral Sciences, University of California, Los Angeles, CA USA; 2 RAND Corporation, Santa Monica, CA USA; 3 Center for Community Health, Semel Institute for Neuroscience and Human Behavior, University of California At Los Angeles, Los Angeles, CA USA; 4 Department of Family Medicine, University of Washington, Seattle, WA USA; 5 Veterans Affairs Greater Los Angeles Health Care System, Los Angeles, CA USA; 6 Center for Technology and Behavioral Health, Geisel School of Medicine, Dartmouth College, Lebanon, NH USA; 7 Center for Clinical Trials Network, National Institute On Drug Abuse, Bethesda, MD USA; 8 Veterans Affairs Puget Sound Health Care System, Seattle, WA USA; 9 Department of Psychiatry and Behavioral Sciences, University of Washington School of Medicine, Seattle, WA USA

**Keywords:** Telemedicine, Opioid use disorder, Medication for opioid use disorder, Rural community, Primary care, Implementation, COVID-19

## Abstract

Telemedicine (TM) enabled by digital health technologies to provide medical services has been considered a key solution to increasing health care access in rural communities. With the immediate need for remote care due to the COVID-19 pandemic, many health care systems have rapidly incorporated digital technologies to support the delivery of remote care options, including medication treatment for individuals with opioid use disorder (OUD). In responding to the opioid crisis and the COVID-19 pandemic, public health officials and scientific communities strongly support and advocate for greater use of TM-based medication treatment for opioid use disorder (MOUD) to improve access to care and have suggested that broad use of TM during the pandemic should be sustained. Nevertheless, research on the implementation and effectiveness of TM-based MOUD has been limited. To address this knowledge gap, the National Drug Abuse Treatment Clinical Trials Network (CTN) funded (via the NIH HEAL Initiative) a study on Rural Expansion of Medication Treatment for Opioid Use Disorder (Rural MOUD; CTN-0102) to investigate the implementation and effectiveness of adding TM-based MOUD to rural primary care for expanding access to MOUD. In preparation for this large-scale, randomized controlled trial incorporating TM in rural primary care, a feasibility study is being conducted to develop and pilot test implementation procedures. In this commentary, we share some of our experiences, which include several challenges, during the initial two-month period of the feasibility study phase. While these challenges could be due, at least in part, to adjusting to the COVID-19 pandemic and new workflows to accommodate the study, they are notable and could have a substantial impact on the larger, planned pragmatic trial and on TM-based MOUD more broadly. Challenges include low rates of identification of risk for OUD from screening, low rates of referral to TM, digital device and internet access issues, workflow and capacity barriers, and insurance coverage. These challenges also highlight the lack of empirical guidance for best TM practice and quality remote care models. With TM expanding rapidly, understanding implementation and demonstrating what TM approaches are effective are critical for ensuring the best care for persons with OUD.

## Background

Telemedicine (TM) enabled by digital health technologies to provide medical services has been considered a key solution to increasing health care access in rural communities [1, 2]. However, despite the impact of the opioid crisis on rural communities and the limited resources in these areas to provide effective treatment to all those in need, the use of TM to treat opioid use disorder (OUD) has been limited. Notable implementation barriers include TM regulations (e.g., licensing, reimbursement) and contextual factors inherent to rural communities such as broadband availability. With the immediate need for remote care due to the COVID-19 pandemic, many TM restrictions have been waived, and health care systems have rapidly modified their practice to include digital technologies (e.g., telephone, video chat) for remote primary care and specialty services, including medication treatment for OUD (MOUD). In recent years and even more so now, in concert with the rapid uptake of TM, many articles and commentaries [3, 4] have been advocating for wider and greater use of TM to improve access to health care and have suggested that broad use of TM during the pandemic should be sustained. While increasing access to care through TM for people with OUD, especially in rural areas severely impacted by the opioid crisis is desirable, research on the implementation and effectiveness of TM-based MOUD is limited.

To address this gap in the literature, prior to the COVID-19 pandemic, we conceived of a study designed to address the need for greater access to MOUD by incorporating the option for TM services delivered by an external MOUD provider for patients in rural primary care clinics. Since the pandemic, TM uptake has been rapid, including the use of TM for primary care and, in some rural clinics, TM for MOUD. Even with changes in patient flow in healthcare systems and the unexpected, abrupt introduction of TM in these settings, the need to study the feasibility and effectiveness of TM for MOUD remains. Thus, despite changes in the TM policy and implementation landscape due to the pandemic, we began the feasibility phase of our study. In this article, we share our experiences, including some of the challenges we encountered, during the initial period of the feasibility study. We expect some of these challenges will be overcome later in the study and perhaps after. Nevertheless, there is an immediate need to understand early challenges, given the unprecedented rapid changes in models of healthcare delivery due to the pandemic and a number of clinics beginning to implement TM without implementation guidance or evidence about effectiveness.

## Main text

### The Project: Rural Expansion of Medication Treatment for Opioid Use Disorder

MOUD approved by the U.S. Food and Drug Administration (FDA) are effective and indeed life-saving, but despite national efforts to increase availability through office-based opioid treatment (OBOT) programs, uptake remains slow. Rural communities are of particular concern, as they suffer disproportionately from the opioid crisis [5, 6], but have limited access to MOUD services. In 2015 the overdose death rates for rural areas surpassed the death rate for urban or suburban areas [7]. People living in rural areas face a number of barriers in accessing MOUD, including a limited number of clinics that provide MOUD, a shortage of providers who prescribe opioid treatment medications, long distances to travel to their opioid treatment providers, and stigma associated with opioid use disorder treatment-seeking in local communities [1]. Additionally, economic distress, older populations, and social isolation play significant roles in opioid addiction in rural communities [8, 9]. There is a need to identify and study effective ways to expand treatment access and improve retention on MOUD in highly impacted rural areas. TM-based MOUD offers an additional option to the traditional OBOT to increase access to care.

In responding to the opioid crisis in rural communities, the National Drug Abuse Treatment Clinical Trials Network (CTN) funded (via the NIH HEAL Initiative) a study on Rural Expansion of Medication Treatment for Opioid Use Disorder (Rural MOUD; CTN-0102). The study aims to investigate the implementation and effectiveness of adding TM-based MOUD to rural primary care clinics. This 5-year study has two phases. The first phase is a feasibility study intended to develop and pilot test the study procedures and evaluate the feasibility and preliminary outcomes of a care coordination model that adds TM-based MOUD from a provider external to the rural clinic by supporting referral and coordination between the clinic and TM provider. The second phase is a large-scale pragmatic trial to test the effectiveness of this model. In this commentary, we present early experiences, including challenges in implementing TM-based MOUD during the first two months of a 6-month pilot test. The challenges discussed in this article were observed through the research team’s interaction with rural primary care clinics and the study TM provider (a vendor outside of the clinics) during recruitment of potential study clinics and with the selected study clinics during regular quality assurance (QA) meetings in the first 2 months of the study. During the clinic site recruitment phase, the study team visited potential sites to discuss study participation with clinic leadership, providers, and other staff. Additional clinics were recruited after televideo or telephone interviews. After study start-up, QA meetings were conducted with participating study clinics weekly and involved different staff members when needed and available, most often the clinic’s care coordinator assigned to facilitate the study, clinic MOUD champion, and/or information technology (IT) staff. The purpose of these weekly QA calls was to ensure protocol adherence and identify challenges, given that their identification can suggest quick adjustments in the implementation of TM. A more formal qualitative analysis of attitudes toward MOUD treatment and TM that includes interviews and focus groups with staff as well as patients with OUD across all clinics is currently underway.

### The care coordination model between the tm provider and rural primary care clinic

Primary care is at the core of rural health care systems. Most rural primary care clinics are in medically underserved areas, facing a workforce shortage and high demand for care [8–10]. To quickly expand OUD treatment access in rural areas, the study intervention is designed to facilitate cooperative relationship between an established, independent TM vendor and the rural primary care clinics that may or may not have an existing OBOT program [1]. By integrating referral and coordination procedures with an established TM vendor into clinic workflows, rural health centers may be able to quickly extend their capacity to serve more individuals with OUD in their community, which can lead to improved quality of patient care and healthier communities.

The study team partnered with an established TM vendor that could work closely with clinics on referrals and provide MOUD and behavioral health services (e.g., individual or group sessions) as needed. This partnership allows for rapid study startup, standardized TM delivery, and potential scale-up if positive results are obtained. At the time the study was conceived (prior to COVID-19), using on-site, credentialed clinic providers to provide MOUD via TM was often not feasible due to limited TM infrastructure and waivered providers. Although many TM restrictions are temporarily in abeyance during the national emergency caused by COVID-19, clinics still experience limited resources for MOUD that likely are not going to change as quickly as needed to address the ongoing opioid crisis.

The TM vendor chosen for the study provided TM services in 24 states at the time the project started. The vendor provides a comprehensive TM-based program using video conferencing between patients and their clinicians for medication prescription and management, behavioral therapies, and remotely viewed saliva/urine drug screens. Most importantly, the vendor is flexible in providing services that meet clinics’ and patients’ needs. The study does not dictate or require clinics to refer all their OUD patients to the TM vendor but instead encourages collaborative efforts to provide services that best suit patients’ needs. To ensure successful collaboration, a service delivery protocol was developed between the TM vendor and each clinic to cover the terms of service, including the following: the services desired by the clinic from the TM provider (e.g., medication treatment for OUD, behavioral health services for OUD, coverage as needed when on-site clinicians are not available), referral options (e.g., warm handoff, online referral, faxed referral), ways to communicate about patient progress (e.g., regular clinical updates, direct messaging to EHR, conventional calls), and plans to address no-shows and dropout from services. Clinics refer patients to the TM vendor just as they would for other external services; this means the TM vendor bills their clinical services directly to relevant insurance/coverage, allowing each organization to maintain their independence with separate finances and budgets.

### The feasibility study

Seven rural primary care clinics in three states (Maine, Washington, Idaho) participated in the feasibility phase of the study. We selected clinics with varying levels of OBOT capacity (i.e., according to the number of buprenorphine-prescribing clinicians at the clinic: 0, 1–3, or more than 3) to maximize the opportunity of observing diverse implementation patterns and issues that can inform implementation procedures for clinics participating in the pragmatic trial. These findings will have implications for the larger, pragmatic trial phase of the study with respect to how an optimal care coordination model with TM referral and coordination can be shaped or achieved. Despite the onset of COVID-19 placing tremendous demands on the clinics and their staff, they completed all training needed for study implementation in just 8 months. Training topics included study protocols, data safety, research ethics, and OBOT, as well as preparation tasks to set up study-related procedures and establish connections with research teams and the TM vendor. The first clinic started the feasibility study in July 2020; by August 2020 the remaining clinics had begun. The feasibility study lasted for 6 months for each clinic.

### Clinics’ motivation for study participation

The study team conducted site visits to potential clinics during the site recruitment period (pre-COVID-19) to present and discuss the study and to address questions from the clinic leadership and staff. Example issues regarding TM that arose during site visits include assurance of compatibility of TM with their treatment philosophies (e.g., whether TM is a patient-centered approach and whether TM supports harm reduction or if abstinence is the goal), development and maintenance of remote patient-provider relationships, as well as resolving perceived or potential competition over patients with the TM vendor. Motivations for study participation included the need to address capacity limitations, including a limited number of on-site prescribers with a Drug Enforcement Administration X-waiver to prescribe buprenorphine, lack of or limited access to behavioral health services, need for additional help to address the needs of non-adherent or more complex patients, and a desire to maximize options of care for patients, particularly when their needs exceeded the limitations of a physical facility (i.e., transportation, childcare, or work issues that prevent patients from maintaining appointments in the clinic). Another motivation mentioned by some clinics was that moving some of their patients to the study TM vendor would allow them to see patients who had been on their waitlist for months for OUD treatment or behavioral health services. One clinic that already had many X-waivered providers still welcomed the opportunity to incorporate TM into the clinic for the purpose of expanding capacity to serve additional people with OUD from surrounding communities that may have logistical challenges accessing care.

### EarlyChallenges

During the first few months of the feasibility study, we observed multiple challenges, including low rates of identification of new patients with OUD through screening, low rates of referral to TM, digital/internet access issues experienced by patients, challenges with TM referral and vendor capacity, and insurance coverage.

#### Low rates of identification of new patients with OUD from universal screening; challenges with OUD diagnosis

To identify patients with OUD who could benefit from MOUD, participating clinics were asked to screen for OUD among all adult patients at least once during the six-month feasibility study period. TAPS (2–3 items for opioid use) [11] and the DSM-5 checklist for OUD (11 symptoms) [12] were recommended by the study for screening and diagnosing OUD, respectively, if clinics did not have other tools in use. Most clinics used the full TAPS except for two that used DAST-10 [13] and another that used the ASSIST [14]. All clinics used DSM-5 criteria for OUD diagnosis. During the first few months of the feasibility study, screening yielded few participants. In fact, of more than four thousand patients screened, except for self-referred patients, no new patients were identified with a positive OUD screen. This finding is surprising in light of other studies which have suggested an approximately 1% prevalence of OUD among primary care populations [15].

Clinics participating in the feasibility study varied in their experience of screening, diagnosing, and treating individuals with OUD. Among clinics that started universal screening specifically for the study, some reported that a substantial proportion of primary care patients declined an OUD screen, and care coordinators in these clinics relayed patients’ complaints or dissatisfaction with being asked sensitive questions as well as frustration with repeat screens. During the early days of the feasibility study, some primary care providers expressed confusion regarding the process of OUD diagnosis, such as being unsure about how to follow-up with patients who screen negative despite clear risk for opioid misuse observed clinically. Some also conveyed that even when providers suspected patients may have unhealthy use of opioids, some providers were uncomfortable discussing OUD diagnoses with patients in anticipation of a negative reaction from patients. Some clinicians also reported that some patients on long-term opioid therapy did not want an OUD diagnosis recorded in their medical records. These challenges suggest that rural clinicians may need more support or training to be comfortable approaching or starting conversations with patients exhibiting opioid use problems, which is similar to what has been found with primary care physicians broadly [16, 17].

Challenges implementing new interventions into primary care generally are well-documented (i.e., complexity of the intervention, ability to visualize how the new practice fits into the clinic workflow, having enough time to implement the practice, self-efficacy to implement the practice) [18]; thus we can expect that at least some of these challenges may be mitigated over time. While additional clinical training and support may help increase the yield of screening as well as improve providers’ interactions with patients with OUD, other barriers, such as stigma toward patients with OUD, likely will persist. Addressing substance use stigma in the community and healthcare settings and incorporating additional strategies that can reach individuals with OUD are vital steps to efficiently identifying patients with OUD and linking them to MOUD. In addition to support or training to improve provider comfort, part of addressing stigma can include supports to improve patient comfort discussing opioid use and OUD with their healthcare providers.

#### Low TM referral rates

Over the first two months of the feasibility study, TM referral was low, with only 6% of approximately 450 patients with diagnosed OUD being referred. While one clinic with no X-waivered providers referred all patients with OUD (once identified) to TM, few new patients with OUD were identified in this clinic. Moreover, there were variations regarding which patients clinics chose to refer to TM. For example, TM referrals by one clinic with an established OBOT program and many X-waivered providers were only made for clinically complex patients (e.g., those not adhering to medication instructions). Some clinics referred patients to the TM vendor for behavioral health services only, and MOUD was still managed by the local clinic’s providers. Among clinics that were offering the TM option to all of their patients with OUD, the majority of patients did not accept the TM referral. One provider conveyed that some of their patients may consider the opportunity to take a trip to attend clinic visits (as opposed to staying at home) an important social activity.

Clinic champions and coordinators responsible for implementing TM perceived that low referral rates might be related to lack of trust in the effectiveness of TM for treatment of OUD and concerns that patients may have suboptimal adherence to online therapy appointment sessions. Rural residents have been characterized as generally distrusting outsiders [8], and patients may particularly have trust issues with an outside TM provider, as opposed to distrust TM generally. These patients have created strong relationships with their clinic providers and being sent to a new unknown online company may have caused hesitation. Some care coordinators also perceived that the lack of a private place at home to attend appointments would prevent patients from engaging in TM sessions. As reported by one care coordinator, several patients with OUD under drug court were required to attend in-person services by their drug court judge. The care coordinator reported an initial lack of support for MOUD in primary care by the courts and now, with the introduction of TM, skepticism about the appropriateness of TM for court-referred patients. Season of the year was also reported as a possible reason for low referral rates, as providers reported that patients are more active in outdoor activities in the summer months. These clinics speculated an increase in demand for TM services will occur in the winter months (for those with internet connectivity at home) because getting to the clinic will be harder.

#### Digital device and internet access challenges

TM services require internet access and a device on which a video application can be used but, according to not only clinic providers and staff but also the TM vendor, many patients in rural areas do not have adequate access to the internet or the needed mobile device with adequate service data allowance that can support the use of TM. The TM provider noted anecdotally that the digital access problem was particularly worse when public WIFI sources (such as coffee shops and libraries) were closed during the COVID-19 pandemic. One clinic also reported that many of their patients are having trouble with the tablet (available in the clinic) for screening because they have never used a touch screen device.

Lack of digital access could have implications for disparities in access to TM-based care. In one of our prior studies [19] based on the 2019 national survey conducted by the Census, we found that disproportionally higher rates of poor and racial/ethnic minorities (particularly American Indian or Alaska Native, black, and Hispanic) in rural communities lacked either computers or smartphones with internet connections. In addition, gaps in technical skills to navigate various online platforms could also become a roadblock for TM uptake, especially among patients with limited exposure to, or experience with, technology. Although federal TM policy has focused on provider reimbursement and clinicians’ capacity to deliver care remotely, patients’ lack of internet connectivity, appropriate devices, and digital skills remain problematic and, if unaddressed, may lead directly to even greater health disparities, noticeably among those poor or racial/ethnical minorities who already face many other disparities.

#### Workflow and capacity barriers

Despite efforts to assist clinics in developing standard operating procedures (SOP) that provide step-by-step descriptions and visualizations for how screening, diagnosis and TM referral could be incorporated into clinic workflows, some clinics in this early stage still encountered difficulty implementing the new practices. Throughout the first two months of the feasibility study, clinics adjusted their workflows and procedures. For example, one clinic started by having front desk staff conduct OUD screening in the lobby and later determined that having a nurse conduct the screening in an exam room would be more comfortable for patients. This change may have the potential to yield more positive screens. Two other clinics reported plans to schedule patients to come in 15 min prior to their appointments to complete screening.

There were also workflow and capacity challenges to referral and coordination with the TM vendor during early implementation. For example, some clinic staff were unclear about TM referral procedures, perhaps suggesting the need for further training on new workflow procedures among the partner organizations. In addition, the virtual handoff process usually requires a private room in the clinic, adding burden to some clinics’ space management. Several clinics experienced long, unanticipated wait times to set up an initial appointment for referred patients with the TM vendor. During the COVID-19 pandemic, the study TM vendor’s service capacity was further challenged by an influx of patients with mental health symptoms and alcohol use. Additionally, many clinic staff were working remotely, and some were furloughed, which also impacted early implementation. At the start of COVID-19, most clinics limited in-person visits and conducted remote telephone or televideo visits. One clinic reported that although in-person visits have gradually resumed in their clinic, social distancing continues to impose challenges to clinic workflows, including implementation of screening and TM referral.

#### Insurance coverage variability

To ensure TM can be covered by insurance including Medicaid, the study requires that clinics accept Medicaid and/or are in Medicaid expansion states. The study TM vendor accepts almost all forms of insurance including Medicaid. Nevertheless, there are many local Medicaid carriers, and not all cover services provided by the study TM vendor. One patient referred by a study clinic was determined by the TM vendor to be “financially ineligible” because the patient’s Medicaid provider did not cover the study TM vendor. Study investigators reached out to the local authorities and facilitated the establishment of a TM contract with that particular insurance provider. After this experience, team members discovered the same was true of a second Medicaid carrier and repeated this process. Nevertheless, the complication of local variations in insurance coverage for TM adds further challenges to TM access.

## Conclusions

TM offers options and solutions to many barriers to OUD care that rural communities face. The current study aims to test a care coordination model based on referral and coordination between an external TM vendor and primary care clinics; study design and procedures introduce new workflows for identifying individuals with OUD, referring to TM, and tracking and documenting these procedures. The early experiences and challenges identified are largely related to establishing a new service relationship (e.g., referral process), implementing new study procedures (e.g., screening, diagnosis), as well as structural barriers (digital access, insurance), particularly in the context of the COVID-19 pandemic. As noted, some of these challenges are clinic-specific, as these clinics were selected, by design, with diverse characteristics (e.g., West vs. East coast location, number of prescribers).

Despite the challenges identified thus far, clinics participating in the feasibility study and the TM vendor are working to address these barriers. We have observed that many clinics now offer clinic space and devices for use by patients lacking personal access. Some clinics are adjusting their screening procedures to provide greater privacy as well as opportunities for questions and answers with clinic staff regarding screening. Other clinics have spearheaded community outreach to attract more patients with OUD to seek MOUD. The research team will continue to encourage/support clinics in community outreach activities, such as advertising TM accessibility at community centers, churches, substance use specialty care settings, and emergency departments, as well as promoting the study via neighborhood social network applications (e.g., NextDoor, Ring). The study team is also planning additional provider training and technical assistance to ease the referral process, diagnosis challenges, and engaging patients in OUD treatment and TM. Developing trust between clinics and an external TM vendor is also essential for a successful care coordination model, and this will take time and frequent communication. The same is true for adding new procedures to clinical workflows and implementing a new study.

**Fig. 1:**
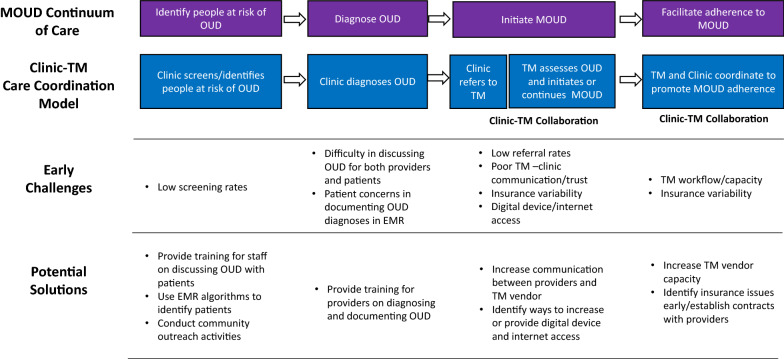
Primary care-telemedicine (TM) care coordination model for treating people with OUD: early challenges and potential solutions

We summarize in Fig. 1 the different points in the OUD care continuum addressed by the care coordination model, barriers experienced, and potential responses based on preliminary lessons learned. Many of these challenges may not be specific to rural communities, but appear to have been exacerbated by vulnerabilities unique to rural areas (e.g., digital access, social distress and isolation) described earlier. Nevertheless, clinics and providers participating in the feasibility study demonstrated a strong commitment to serve their patients and communities, which is solid ground upon which to continue to improve treatment for people with OUD.

As noted, many TM restrictions have been waived due to COVID-19. There are efforts [20] underway to allow these waivers to continue even after the public health emergency ends. Therefore, the movement to expand telemedicine is anticipated to grow even faster and more widely in the near future. Nevertheless, while there have been some non-experimental studies supporting the effectiveness of TM [21], there still have been no randomized controlled trials demonstrating the effectiveness of TM vs. in-person or other control conditions for treating OUD. This is an area in which expansion of services or practices outpaces research, either by necessity (e.g., COVID-19) or logical evolution (logistical convenience).

There are many questions remaining as to best practices for including TM-based MOUD in primary care. For example, which patients are appropriate for TM referral? What is the best way for provider-patient remote relationships to be developed and maintained? What is the best model for TM? The current study assesses a care coordination model that includes referral and coordination of TM in primary care, but there are many other ways that TM can be delivered. In addition to convenience and flexibility in care choices, empirical investigations are needed to identify remote care models that meet the variety of needs among patients for optimal care.

In this commentary, we highlight our experiences and challenges in the initial phase of our study, based on our observations interacting with the rural primary care clinics, with the hope of stimulating more questions and investigations to improve the study and implementation of evidence-based TM care. The research team is currently conducting focus groups and phone interviews with clinic leadership, providers, and staff as well as patients, in order to more systematically identify and understand barriers and facilitators of implementing TM in primary care clinics. This line of inquiry should lead to a better understanding of efficient implementation and delivery of quality TM care. With TM expanding rapidly, understanding implementation and proving effectiveness are critical for ensuring the best care for people with OUD.

## Data Availability

Not applicable.

## Abbreviations

ASSIST: Alcohol, smoking and substance involvement screening test
COVID-19: Coronavirus disease 2019
CTN: The National Drug Abuse Treatment Clinical Trial Networks
DAST: Drug abuse screen test
DEA: Drug Enforcement Administration
DSM-5: Diagnostic and Statistical Manual of Mental Disorders 5th Edition
FDA: Food and Drug Administration
HEAL Initiative: Healing to End Addition Long-term Initiative
MOUD: Medication treatment for opioid use disorder
OBOT: Office-based opioid treatment
OUD: Opioid use disorder
QA: Quality assurance
Rural MOUD/CTN-0102: Rural expansion of medication treatment for opioid use disorder
SOP: Standard operation procedures
TAPS: Tobacco, alcohol, prescription medication, and other substance use
TM: Telemedicine
TREATS Act: Telehealth response for E-prescribing Addiction Therapy Service Act
